# The Amendment of the Medical Acts

**Published:** 1901-06-29

**Authors:** 


### THE AMENDMENT OF THE MEDICAL ACTS.

The object of the Medical Acts Amendment Bill which has been introduced into the House of Commons is to give to the General Medical Council power to erase from the Medical Register for a limited period the name of a registered practitioner who has been adjudged guilty of misconduct in a professional respect, thus removing from the Council one great disability under which it now labours in maintaining discipline, namely that it can only take cognisance of one crime—infamous conduct; and can only mete out one punishment—removal from the register.

